# Heavy Metals Assimilation by Native and Non-Native Aquatic Macrophyte Species: A Case Study of a River in the Eastern Cape Province of South Africa

**DOI:** 10.3390/plants10122676

**Published:** 2021-12-06

**Authors:** Getrude Tshithukhe, Samuel N. Motitsoe, Martin P. Hill

**Affiliations:** Centre for Biological Control, Department of Zoology and Entomology, Rhodes University, P.O. Box 94, Makhanda 6140, South Africa; s.motitsoe@ru.ac.za (S.N.M.); m.hill@ru.ac.za (M.P.H.)

**Keywords:** bio-concentration factor, enrichment factor, geo-accumulation index, metal contamination, phytoremediation, water quality

## Abstract

There is continuous deterioration of freshwater systems globally due to excessive anthropogenic inputs, which severely affect important socio-economic and ecological services. We investigated the water and sediment quality at 10 sites along the severely modified Swartkops River system in the Eastern Cape Province of South Africa and then quantified the phytoremediation potential by native and non-native macrophyte species over a period of 6 months. We hypothesized that the presence of semi and permanent native and non-native macrophytes mats would reduce water and sediment contamination through assimilation downriver. Our results were variable and, thus, inconsistent with our hypotheses; there were no clear trends in water and sediment quality improvement along the Swartkops River. Although variable, the free-floating non-native macrophyte, *Pontederia* (=*Eichhornia*) *crassipes* recorded the highest assimilation potential of heavy metals in water (e.g., Fe and Cu) and sediments (e.g., Fe and Zn), followed by a submerged native macrophyte, *Stuckenia pectinatus*, and three native emergent species, *Typha capensis*, *Cyperus sexangularis,* and *Phragmites australis*. Pollution indices clearly showed the promising assimilation by native and non-native macrophytes species; however, the Swartkops River was heavily influenced by multiple non-point sources along the system, compromising the assimilation effect. Furthermore, we emphasise that excessive anthropogenic inputs compromise the system’s ability to assimilate heavy metals inputs leading to water quality deterioration.

## 1. Introduction

Aquatic ecosystems have been subjected to organic and inorganic pollution, which have worsened with poor waste water management [1]. These impacts have resulted in a noticeable loss of aquatic biodiversity, water quality deterioration, ecosystem integrity, and important socio-economic services [2]. Therefore, effective rehabilitation practices and conservation strategies are needed to minimize and control freshwater contamination.

Previous field and mesocosm trials have shown that reversing the impact of anthropogenic inputs in the environment is challenging, and that minimising the level of these inputs and waste management will help curb environmental contamination [3]. Ecologists have tested different methods to try and reduce contamination in freshwater systems, and these include adsorption, soil washing, reverse osmosis, coagulation, and flocculation [4,5,6].

However, Hanif et al. [7] showed that these methods were costly, sometimes ineffective, and disruptive; for example, soil washing alters sediment microbial communities making it difficult to re-use the treated soil [8]. Methods, such as ion-exchange and artificial membranes, generate end-waste material that requires special deposition, thus, creating additional costs for their disposal [9], whilst coagulation and flocculation can be ineffective in decolorizing laundry effluents [10].

It is clear that there is a need for innovative techniques with merits over traditional methods. Green technology, such as phytoremediation, which uses plants and associated microbes to assimilate and breakdown contaminants in natural environments, is one method that has been widely researched and applied [2,11,12,13,14,15,16]. Phytoremediation is the most innovative, cost-effective, and environmentally friendly technology available to assimilate organic and inorganic contaminants even at low concentrations [12,17,18]. Studies have shown that phytoremediation has socio-economic and environmental merits over traditional physicochemical clean-up, and can reduce water quality contamination by more than 50% in mesocosm settings [19,20,21,22,23,24].

The assimilation efficacy of macrophytes has been studied by several researchers [9,25,26,27,28,29,30,31]. These studies investigated the fate of toxic and non-toxic elements in the field and laboratory using native and non-native macrophytes, and each case study showed improved water chemistry, through reduced nutrients, and heavy metal concentrations after assimilation. To date, phytoremediation feasibility studies have focused on the treatment of heavy-metal contamination when using macrophytes species, such as *Typha capensis* (Rohrb.) N.E.Br. (Typhaceae) (Bulrush), *Phragmites australis* (Cav.) Trin. ex Steud (Poaceae) (Common reed), and *Cyperus sexagularis* (L.) (Cyperaceae) (Swamp flat-sedge) [32,33,34,35]. These macrophytes species are widespread and abundant in freshwater systems, they can tolerate different environmental constraints, thus, making them significant candidates for phytoremediation [30]. Furthermore, these macrophytes provide basic ecosystem services that serve an important role in biogeochemical processes, the natural cycling of nutrients [36], and supplying the system with a continuous source of energy [37].

Similarly, non-native macrophytes, including *Salvinia molesta* D.S. Mitchell (Salviniaceae) (Giant Salvinia), *Pistia stratiotes* L. (Araceae) (Water lettuce), and *Pontederia crassipes* (Mart.) Solms-Laub. (Pontederiaceae) (Water hyacinth), have shown to be excellent bio-accumulators [17,31,38,39]. These non-native macrophytes species are natural hyper-accumulators and can be effective in assimilating pollutants more than native macrophytes in their introduced range [12,40,41]. Their fibrous root systems, high biomass production rates, and tolerances to disturbed and heavily polluted systems justify their use in treating wastewater, and improving the water quality by assimilating different metals, such as Zinc (Zn), Arsenic (As), Lead (Pb), Chromium (Cr), and Manganese (Mn) (as seen in Ali et al. [12]).

The selection of plant species for phytoremediation is usually based on their tolerance and ability to accumulate a wide range of contaminants [42]. Non-native macrophytes thrive extremely well in phosphate and nitrate enriched waters as compared to native macrophytes in South Africa [43]; however, such conditions promote high biomass of non-native macrophyte species, such as *S. molesta*, *P. stratiotes,* and *P. crassipes*, making them more effective accumulators, but more also invasive, and thus displacing native aquatic biodiversity [42,44].

Secondly, although non-native macrophyte species have proven to be better assimilators of heavy metals [16,40,41]. Non-native macrophytes are equally destructive they modify invaded ecosystems by altering the hydrology and aquatic species composition, reduce ecosystem processes, production, and contribute to lose of aquatic biodiversity [1,16,45,46]. Therefore, in this study, we field test the assimilation potential of both native and non-native macrophyte stands found along the Swartkops River in South Africa. We hypothesize that the presence of native and non-native macrophytes species will help reduce the heavy metal contamination in water and sediments downstream of semi and permanent native and non-native stands.

## 2. Materials and Methods

### Study Area

**Figure 1:**
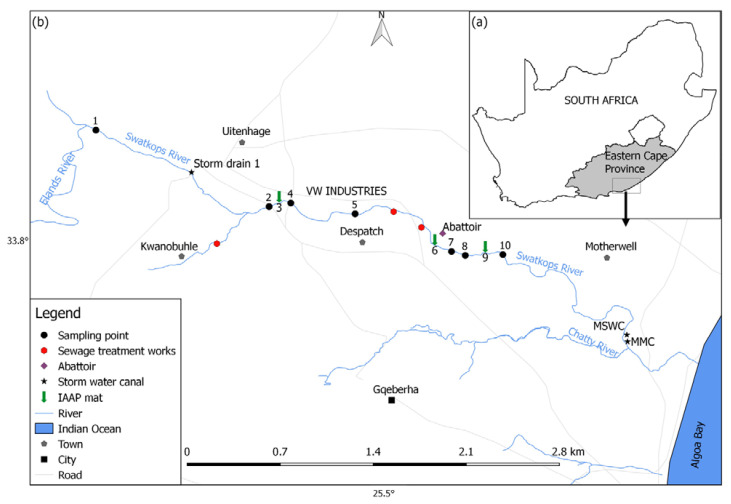
The South African map insert (**a**) the Swartkops River and tributaries showing 10 sampling locations and land-use activities along the river system (**b**) Motherwell Storm Canal (MWSC) and Markman Canal (MMC).

The study was conducted in the Swartkops River (33°45′08.0″ S 25°20′33.1″ E to 33°48′37.50″ S 25°30′46.80″ E), Uitenhage, Eastern Cape Province of South Africa. The Swartkops River and its tributaries, i.e., KwaZunga and Elands rivers, arise in the Groot Winterhoek Mountains of the Swartkops catchment and flow into Algoa Bay, and into the Indian Ocean (Figure 1) [47]. The Algoa Bay is an important coastal line in South Africa, known for its marine biodiversity and serving as a habitat and nursery site for various marine animals, including *Spheniscus demersus* (African penguins), *Mirounga leonina* (Southern elephant seal), and *Sphyrna zygaena* (Great white shark) [48].

The 155 km long Swartkops River drains the 42 km^2^ wide catchment area where protected areas dominate the upper reaches of the catchment; the middle reaches is dominated by urban, formal settlements, and agricultural lands; and the lower reaches are surrounded by industries, formal and informal settlements before flowing into the ocean. The landscape activities contribute to the release of domestic effluents, industrial waste, untreated sewage, and other point and non-point source pollutants [49]. The natural vegetation dominating the lower catchment is Bushveld and Succulent thicket, which has been severely altered by the introduction of alien invasive plant species, such as *Eucalyptus* spp. (Gum trees) and *Acacia* spp. (Black Wattle and Port Jackson Willow) [49].

Ten study sites were selected along the Swartkops River and sampled for a period of six-months, at monthly intervals, from April 2018 to September 2018. Sample collection took place upstream and downstream of semi and permanent non-native macrophytes mats, *P. crassipes* and *S. molesta* (Figure 1). Site 1 was situated among agricultural lands, which was upstream from Uitenhage town but downstream from protected areas. The site experienced minimal urban and industrial effluents except some agricultural inputs (Figure 1). Site 2 was situated downstream from site 1, in the heart of the Uitenhage urban area and after the confluence of Swartkops River and KwaNobuhle tributary. Site 2 was less than 1 km upstream from *P. crassipes* mat 1 (hereafter site 3), whereas site 4 was located ~0.6 km downstream from site 3 (Figure 1). Site 5 was 2.4 km upstream from *P. crassipes* mat 2 (hereafter site 6), and site 7 was about a kilometre downstream from site 6 (Figure 1). Site 8 was located ~1.6 km upstream from *S. molesta* mat 3 (hereafter site 9), and site 10 was located 0.6 km downstream from site 9 (Figure 1).

At each site, water and sediment samples together with dominant native (i.e., *T*. *capensis*, *P*. *australis*, *C*. *sexagularis*, and *S. pectinatus*) and non-native (i.e., *P. crassipes* and *S. molesta*) macrophyte species were collected and analysed for heavy metal accumulation analysis (Table S1).

## 3. Data Collection

### 3.1. Water Chemistry

Integrated water sample (1000 mL, *n* = 1) was collected ~20 cm below the water surface at each site using pre-rinsed clear polyethylene sample container for water chemistry analysis. Water samples were then stored on ice until they reached the laboratory, and, within 48 h after collection, water samples were sent to BEM-Labs, Cape Town, South Africa for water chemistry analysis, including Chemical Oxygen Demand (COD), Zinc (Zn), Iron (Fe), Cadmium (Cd), Arsenic (As), Chromium (Cr), Lead (Pb), Mercury (Hg), and Copper (Cu).

At the laboratory (BEM-Labs), the water samples were acidified to a pH of ±2 and digested to isolate all the metal ions in solution. Once cooled, the samples were filtered through a 0.45 µL syringe filter to remove any particulates. The resulting samples were then analysed using an Agilent ICP-OES 720 Axial instrument for total heavy metals. Since these were integrated water samples, it is possible that some properties, such as pH, or organic carbon, varied between the samples and may have influenced the speciation (and bioavailability) of pollutants.

### 3.2. Sediment Chemistry

Using a gardening trowel, integrated soil sediment samples were collected at five areas per site at approximately 10 cm depth. Sediments samples were collected into plastic zip-lock bags and then stored on ice. Similar to the water chemistry samples, sediment samples were within 48 h after collection sent to BEM-Labs for sediment chemistry and heavy metal analysis, including Zn, Fe, Cd, As, Cr, Pb, Hg, and Cu.

At BEM-Labs laboratory, a portion of the sediment sample was weighed into an Erlenmeyer flask. We added 20 mL nitric acid and 10 mL hydrogen peroxide to the flask, and the flask was then heated to allow the sample to digest. After digestion, the sample was transferred to a 100 mL volumetric flask, made up to volume, and then filtered. The resulting sample was then analysed on the Agilent ICP-OES 720 Axial instrument for heavy metals.

### 3.3. Macrophytes Chemical Analysis

Native marginal and aquatic vegetation species together with non-native macrophytes were collected at each site for heavy metal analysis. Five stems of emergent plants i.e., *T. capensis*, *C. sexangularis* and *P. australis*; five matured floating plants i.e., *P. crassipes* and *S. molesta*, and about 200 g of submerged plant i.e., *S. pectinatus*, were collected by hand and rinsed with distilled water to remove any debris and periphyton biofilm.

Plant material were transferred into different zip lock bags (per plant species) and stored on ice until they reached the laboratory. In the laboratory, plant samples were immediately oven-dried at 60 °C for 72 h. During this procedure, all the cell processes (e.g., respiration) stopped, making sure that samples represents the nutrients composition per gram of leaf without the influence of water. Thereafter, dried leaves were homogenised into coarse material by grinding using a mortar and pestle.

About 6.5 g of dried plants tissue was weight and packaged into aluminium foil envelopes and also sent to BEM-Labs, for heavy metal analysis, including Fe, Hg, Zn, Cd, As, Pb, and Cu. For each sample, 20 mL nitric acid and 5 mL hydrogen peroxide were added and the flask was heated to allow the sample to digest, until approximately 1 mL of the solution was left. The remaining sample was transferred into a 10 mL volumetric flask, made up to volume using distilled water and filtered. The filtered sample was analysed on the Agilent ICP-OES 720 Axial instrument for heavy metals.

## 4. Data Analysis

To assess the reduction in water and sediment chemistry between upstream and downstream semi and permanent *P. crassipes* and *S. molesta* mat sites, the percentage reduction in water and sediment heavy metals concentrations were computed. Furthermore, to understand the current environmental condition at Swartkops River and the concentration of heavy metals, sediments and macrophyte indices were used to quantify heavy metal assimilation by both native and non-native macrophytes along the river system.

The geo-accumulation index (Igeo), which measures the degree of heavy metal contamination, was used to estimate heavy metal pollution in the Swartkops River during the study, and this was calculated following the equation defined by Muller [50]:

$$\mathrm{Igeo}=\log2\;\left(\frac{\mathrm{Cn}}{1.5\mathrm{Bn}}\right)\;$$

where Cn is the measured concentration of metal in sediments, and Bn is the measured geo-chemical background value of the metal. The 1.5 factor is used to minimize possible variations of the background values, which may be qualified to lithogenic variations [51]. The geo-chemical background values were given according to the world surface rock average as seen in Martin and Meybeck [52].

For further geo-accumulation interpretation, Muller [53] proposed seven classes for the geo-accumulation index, which are used to determine the level of contamination on soil sediments by heavy metals: Class 0 = Igeo < 0 (uncontaminated); Class 1 = 0 < Igeo < 1 (uncontaminated to moderately contaminated); Class 2 = 1 < Igeo < 2 (moderately contaminated); Class 3 = 2 < Igeo < 3 (moderately to heavily contaminated); Class 4 = 3 < Igeo < 4 (heavily contaminated); Class 5 = 4 < Igeo < 5 (heavily to extremely contaminated); and Class 6 = 5 < Igeo (extremely contaminated).

Secondly, the pollution load index (PLI), which is an important index in evaluating soil sediment quality was used to estimate heavy metal pollution in the sediments. The pollution load index is expressed as the product of the contamination factor (*CF*) of all measured heavy metals on-site and was calculated following a formula adopted from Islam et al. [54]:

PLI=CF1 × CF2 × CF3 ×…….CFn) 1/n

The Contamination Factor (CF) of each metal was computed separately per site using the metal concentration and the background value of the metal (background value from the average shale value) [55], CF was calculated following Atgin et al. [56].

$$\mathrm{CF}=\frac{\mathrm{Cm}\;\mathrm{Sample}}{\mathrm{Cm}\;\mathrm{Background}}$$

where Cm (sample) is the concentration of heavy metal in sediment and Cm (background) is the background value of metals adopted from world surface rock average by Martin and Meybeck [51]. According to Chakravarty and Patgiri [57], the PLI value < 1, indicates no pollution, whilst PLI value > 1, indicates pollution (or deterioration of the sediment).

The enrichment factor (EF) is a more comprehensive assessment of heavy metal contamination [58]. The method is based on normalisation of the measured heavy metal concentration with respect to the reference metal, such as Aluminium (Al) or Fe [59]. For the present study, Fe was used as a reference heavy metal for normalization because, according to Nirmala et al. [60], Fe is redox sensitive under oxidation conditions and constitutes significant sinks of heavy metals in aquatic ecosystems.

Background values used for the present study were given according to the world surface rock average by Martin and Meybeck [52]. According to Chen et al. [61], EF < 1, indicates no enrichment; EF = 1–2, minimal enrichment; EF = 3–5, moderate enrichment; EF = 5–10, moderately severe enrichment; EF = 10–25, severe enrichment; EF = 25–50, very severe enrichment; and EF > 50, extremely severe enrichment. These were calculated following Buat-Menard and Chesselet [62]:

$$\mathrm{EF}=\frac{\left[\frac{\mathrm{Cmetal}}{\mathrm{Cnormalizer}}\right]\mathrm{Sample}}{\left[\frac{\mathrm{Cmetal}}{\mathrm{Cnormalizer}}\right]\mathrm{Reference}\;\mathrm{metal}\;\left(\mathrm{Fe}\right)}$$

where Cmetal (sample), is the concentration of the examined heavy metal; Cnormalizer (sample), is the concentration of the normalizer/reference heavy metal (Fe); Cmetal (reference metal), is the concentration of the examined heavy metal in its suitable background or baseline reference material, and Cnormalizer (reference metal) is the concentration of the normalizer heavy metal (Fe) in its suitable background.

Then, to assess and estimate the native and non-native macrophyte species accumulation potential for heavy metal concentration in sediments, the bio-concentration factor (BCF) was calculated following Zayed et al. [63]:

$$\mathrm{BCF}=\frac{\left[\mathrm{metal}\;\mathrm{plant}\right]}{\left[\mathrm{metal}\;\mathrm{sediment}\right]}$$

where metal (plant) is the concentration of heavy metals in plants, and metal (sediment) is the concentration of heavy metals in sediments. BCF value > 1, indicates that the plant species is a better hyper-accumulator of the heavy metal; whereas, BCF value = 1 indicates that plant species is an accumulator of the heavy metal, and BCF value < 1 indicates that a plant is a better excluder [64].

To test the significant differences in sediment indices (i.e., Igeo, PLI, and EF) between sites and the macrophyte assimilation factor (BCF) for each plant species, the Shapiro–Wilk test for normality and Levene test for homogenous variance were employed. The outcome of the tests revealed that none of the variables were normally distributed (Shapiro–Wilk, *p* < 0.05) nor were the variances homogenous (Levene test, *p* > 0.05). Thus, a non-parametric test, in this case, Kruskal–Wallis analysis of variance, with multiple comparison test was employed. All statistical analyses were conducted in R version 3.6.1 [65], except where specified.

## 5. Results

### 5.1. Water and Sediment Chemistry

Heavy metal concentrations were variable along the Swartkops River with no consistent reduction trend downriver (Table S2). The Fe concentration showed significant differences between sites (*H* = 28.13, *p* = 0.001) with site 1 recording high Fe concentration (1.1 mg/L) and site 10 low concentration (0.09 mg/L) (Table S2). There was significant difference in Zn concentration between sites (*H* = 18.03, *p* = 0.034), the highest Zn concentration (0.12 mg/L) was recorded at site 10, and the lowest Zn concentration (0.02 mg/L) was recorded for all sites except sites 5 and 7 (Table S2).

The COD concentrations were significantly different between sites (*H* = 21.89, *p* = 0.001). The highest COD concentration was recorded at site 5 (57.4 mg/L) and the lowest at site 1 (14.64 mg/L) (Table S2). The As and Cu concentrations were not significantly different, whereas heavy metal, i.e., Cd, Cr, Hg, and Pb, concentrations showed constant values of 0.0021, 0.026, 0.0021, and 0.006 mg/L, respectively, throughout the sampling period (Table S2).

The sediment chemistry results revealed that the Fe (*H* = 24.32, *p* = 0.004), Zn (*H* = 35.75, *p* < 0.001), As (*H* = 17.08, *p* = 0.05), Cr (*H* = 20.39, *p* = 0.016), Pb (*H* = 26.19, *p* = 0.002), and Cu (*H* = 26.46, *p* = 0.002) concentrations were significantly different between sites (Table S3). Fe (1321.25 mg/kg) and As (4 mg/kg) were high at site 1 and low at site 5 (Fe: 220.43 mg/kg) and site 10 (As: 0.27 mg/kg), respectively.

Zn (87.16 mg/kg), Cr (41.12 mg/kg), Cu (5.56 mg/kg), and P (2240.38) were high at site 5, and low at site 10 (Zn: 7.19 mg/kg and Cr: 8.15 mg/kg) and site 7 (Cu: 0.67 mg/kg, P: 281.07 mg/kg) (Table S3). The lead concentration was high at site 3 (21.10 mg/kg) and the low at site 10 (3.67 mg/kg) (Table S3). In general, the sediment chemistry results revealed that site 5 had the highest recorded heavy metal concentrations i.e., Zn, As, Cr, Pb, and Cu (Table S3).

### 5.2. Swartkops River Sediment Contamination

The geo-accumulation index (Igeo) was significantly different for heavy metals i.e., As (*H* = 17.16, *p* = 0.05), Cr (*H* = 19.08, *p* = 0.02), Cu (*H* = 26.47, *p* < 0.001), Fe (*H* = 24.32, *p* < 0.001), Pb (*H* = 26.19, *p* < 0.001), and Zn (*H* = 21.40, *p* = 0.01) at all sites (Table 1). Cadmium (*H* = 8.26, *p* = 0.51) and Hg (*H* = 5.05, *p* = 0.83) were not significantly different at all sites, and showed negative (−) Igeo values, which are indicative of uncontaminated sediments (Table 1).

Geo-accumulation index values revealed that sites were extremely contaminated by Cr, Fe and Zn, recording Igeo values of more than 5. Site 5 recorded the highest Igeo value for Zn (12.16) and Cr (11.06) whereas, site 1 recorded the highest Igeo value for Fe (15.11) (Table 1). All sites were extremely contaminated (Igeo > 5) with Pb and Cr, except site 9 (Pb) and site 10 (Pb and Cr). Arsenic recorded the lowest Igeo values ranging from −0.64, uncontaminated sediments (site 3), to 2.94, moderately contaminated (site 1) (Table 1).

The enrichment factor (EF) revealed that five heavy metals, including As (*H* = 17.08, *p* = 0.05), Cr (*H* = 20.39, *p* = 0.02), Cu (*H* = 26.47, *p* < 0.001), Zn (*H* = 35.80, *p* < 0.001), and Pb (*H* = 26.19, *p* < 0.001), showed significant differences between sites (Table 1). Site 1 recorded high EF values for majority of heavy metals, i.e., As, Cr, Cu, and Hg, whilst site 5 revealed high EF for heavy metals, i.e., Zn, and Pb (Table 1).

Based on the EF values obtained, all sites experienced no enrichment except for site 1, which showed minimal Hg enrichment (Table 1). The PLI values were not significantly different between sampling periods (Kruskal–Wallis ANOVA, *p* > 0.05) (Table 1). All recorded PLI values were below 1, except site 5, which recorded PLI value of 1.10 for the month of April. In general, the month of June recorded PLI > 1 for majority of sites; however, they were all not significantly different.

### 5.3. Heavy Metal Assimilation along the Swartkops River

*Pontederia crassipes* and *Salvinia molesta* semi and permanent mats showed promising heavy metal assimilation, but this varied between sites. However, in some cases, the trend was clear showing heavy metal reduction between upstream and downstream *P*. *crassipes* and *S*. *molesta* mats, thus, indicating possible macrophyte assimilation potential (Table S4).

The Fe concentration in the sediments showed a 46% reduction between the upstream site 2 (967.37 mg/kg) and downstream site 4 (520.02 mg/kg) of *P. crassipes* mat (site 3), whereas Zn concentration showed a total reduction of 57%, 89%, and 65% between site 2 (62.55 mg/kg) and site 4 (26.92 mg/kg), site 5 (87.16 mg/kg) and site 7 (9.65 mg/kg), site 8 (22.4 mg/kg) and site 10 (7.19 mg/kg), respectively (Table S4). Arsenic showed a total reduction of 81% and 83% between site 5 (1.49 mg/kg) and site 7 (0.28 mg/kg) as well as site 8 (1.55 mg/kg) and 10 (0.27 mg/kg), respectively (Table S4).

Chromium showed a 77% reduction between site 5 (41.12 mg/kg) and site 7 (9.50 mg/kg), and Pb showed a 68% reduction between site 5 (19.90 mg/kg) and site 7 (6.45 mg/kg), and 56% reduction between site 8 (8.42 mg/kg) and site 10 (3.67 mg/kg) (Table S4). Mercury was reduced by 59% between site 2 (1.77 mg/kg) and site 4 (0.72 mg/kg) and by 53% between site 5 (1.36 mg/kg) and site 7 (0.64 mg/kg) (Table S4).

Emergent native macrophytes species recorded the lowest bio-concentration factor (BCF) values when compared to both floating and submerged native macrophyte species (Table 2). *Typha capensis* and *Cyperus sexangularis* BCF results were significant between sites for Cu (*H* = 21.11, *p* = 0.01; *H* = 25.39, *p* = 0.002) and Zn (*H* = 37.34, *p* < 0.001; *H* = 38.45, *p* < 0.001) (Table 2). *Typha capensis* and *C*. *sexangularis* showed a BCF value of less than 1 for Cu at all sites; however, for Zn, *T. capensis* recorded a BCF of less than 1 at sites 1, 6, 7, 8, 9, and 10, whereas *C. sexangularis* recorded BCF of less than 1 at sites 1,7, 8, 9, and 10 (Table 2). *Phrgamites australis* showed significantly different BCF values for Zn (*H* = 16.43, *p* = 0.05), at all sites, except for site 5, which showed BCF values of less than 1 (Table 2).

The floating non-native *P. crassipes* BCF results were significantly different between sites for As (*H* = 23.15, *p* < 0.01), Cr (*H* = 23.32, *p* < 0.001), Cu (*H* =24.4, *p* < 0.001), Fe (*H* = 26.94, *p* < 0.001), Hg (*H* = 20.76, *p* < 0.01), and Zn (*H* = 27.7, *p* < 0.001) (Table 2). *Pontederia crassipes* recorded BCF values of less than 1 for Cu and Zn at all sites; whilst As recorded BCF > 1 for site 1 and site 9, and Hg BCF > 1 at sites 6, 7, 9, and 10 (Table 2).

The submerged macrophyte *S. pectinata* BCF results were significantly different for four heavy metals, i.e., Cr (*H* = 27.33, *p* < 0.001), Fe (*H* = 23.64, *p* = 0.05), Hg (*H* = 16.77, *p* = 0.05), and Zn (*H* = 22.08, *p* < 0.01) (Table 2). The heavy metals Fe, Hg, and Zn recorded BCF of less than 1 for all sites, whilst Cr recorded BCF > 1 at sites 6, 7, and 9 (Table 2). The significant BCF values for *S. pectinata* species were in decreasing order of Hg > Zn > Fe > Cr, indicating that *S. pectinata* assimilated Hg more effectively compared to Cr (Table 2).

## 6. Discussion

The present study reports that the Swartkops River system is heavily polluted by various heavy metals. Although our results show some degree of assimilation by native and non-native macrophyte stands, the continuous inputs, i.e., non-point sources, at different entry points along the river system surpass this potential. Research on macrophyte assimilation (or phytoremediation) has mainly been conducted in mesocosm settings, with limited in situ case studies or case studies in the natural environment [23,66,67,68].

The effectiveness of phytoremediation in the reduction of heavy metal concentrations in water and sediment by non-native *P. crassipes* and *S. molesta* was tested in the present study and others (e.g., [19,29,69,70]). Although the results did not show a consistent decreasing trend due to high variation between sites, the present study’s findings still showed promising macrophyte assimilation potential as most sites showed reduced concentrations of heavy metals as hypothesized. The Swartkops River is in a deteriorating state, and these findings have been corroborated by a number of studies before (see [49,71,72,73]), which revealed that intense land-use developments along the Swartkops River catchment and riparian areas have a huge effect on the system’s physical, chemical, and biological well-being.

Findings from the present study revealed that there were few significant reductions in heavy metal concentrations between the immediate upstream and downstream sites within individual non-native macrophyte patch. For example, between site 2 and site 4 upstream and downstream of *P*. *crassipes* mat (site 3), as well as site 8 and site 10 of *S*. *molesta* mat (site 9), we reported more than 45% reduction in heavy metal concentration (i.e., Zn, Cr, As, Pb, and Hg) (Table S4).

Reductions were attributed to the presence of *P*. *crassipes* and *S*. *molesta* mats acting as accumulators for heavy metals from upstream. The above findings corroborated with Mishra and Tripathi [19], whose study reported on the effectiveness of *P. crassipes* in accumulation of Cr and Zn effluents, were *P. crassipes* efficiently assimilated more than 50% of the heavy metal concentration in only 11 days of exposure, further emphasizing the phytoremediation potential of these macrophytes.

It is possible that some of the pollutants were accumulated by sediments. This is because, as contaminants constantly wash off downriver, some slowly settles and gets assimilated in the sediments. Jernström et al. [74] indicated that the nature of sediments in water bodies reflects, to a great extent, the condition of the system as a result of various pollutants in the water; in addition, these sediments may also serve as indicators by revealing the concentration of the pollutants settling in them.

These results were supported by Hadad et al. [75] and Schaller et al. [76], who reported that the top sediment layer, integrated with a low diffusion rate of elements can play a significant role in adsorption and accumulation of heavy metals. Various indices from the present study, including EF, PLI, and Igeo, showed that sediments along the Swartkops River system were moderately to extremely contaminated as a result of pollutants along the river catchment (Table 1).

These findings were more evident at site 5, which recorded the highest heavy metal concentrations (i.e., Zn, As, Cr, Cu, and Pb) in sediments, in addition, the EF and Igeo values were highest for Zn and Pb, revealing extreme sediment contamination by these heavy metals at site 5 (Table 1). This emphasize that the Swartkops River is facing probable environmental pollution especially with heavy metals, i.e., Fe, Cu, Cr, Zn, and Pb.

The bio-concentration factor (BCF) index also showed that *T. capensis*, *C. sexangularis, P. australis* (native emergent macrophytes), *S. pectinatus* (native submerged macrophyte), and *P. crassipes* (invasive floating macrophyte) have promising assimilation potential. Various studies (i.e., [15,77,78,79]) have shown that *T. capensis*, *C. sexangularis,* and *P. australis* are good heavy metal accumulators. The present study, although variable, were consistent with the above mentioned studies revealing that these macrophyte species are great accumulators of various heavy metals.

This was because both studies showed reductions in heavy metal concentrations indicating phytoremediation potential by native and non-native macrophytes. Despite the macrophyte assimilation potential, few hyper-accumulated heavy metals were recorded when compared to what other studies had achieved when using the same macrophytes species [15,78,79] This could be attributed to fact that the present study only used *C. sexangularis, P. autralis,* and *T. capensis* species leaves for heavy metal analysis. Macrophytes assimilate heavy metals; however, their concentrations differ with plant parts or segments. For example, Vymazal and Březinová [80] reported that the assimilation and distribution of heavy metals in above-ground parts differs from below-ground plant parts, and this is because of different physiological absorption mechanisms in plants. Other studies, including Chandra and Yadav [77], Eid et al. [70], Bonanno [81], and Vymazal and Březinová [80], supported these findings by revealing that emergent macrophytes species, including *Phragmites* spp., *Cyperus* spp., and *Typha* spp., usually have similar accumulation trends.

These macrophytes species accumulate larger quantities of certain heavy metals, including Cr, Mn, Cu, Ni, Hg, Pb, and Zn, better in underground plant parts as compared to above-ground plant parts, and this is usually in the order of roots > rhizomes > leaves > stems. Although the present study did not evaluate heavy metal concentrations for below ground plant parts for *P. australis*, *C. sexangularis,* and *T. capensis*, the accumulated heavy metal concentrations and low BCF values recorded in emergent macrophytes could have been influenced by the same trend, which is variation in the distribution within the plant parts, which may also differ with plant size.

In contrast, floating (non-native) and submerged (native) species revealed a greater uptake of heavy metals (i.e., Cr, Fe, Hg, and Zn) with high BCF values compared to emergent macrophytes (Table 2). This was expected for *P. crassipes,* as it is known for a high accumulation ability and tolerance to disturbances. The high uptake of heavy metals by *S. pectinatus* could have been solely influenced by using the whole plant (roots, stem, and leaves), which were fully exposed to heavily polluted systems.

The present study further revealed that *P. crassipes* was the most effective accumulator of heavy metals, followed *S. pectinatus*, *P. australis*, *C. sexangularis,* and *T. capensis*. The order of accumulation in heavy metals by macrophyte species (floating, emerged, and submerged) was similar to a study by Goulet et al. [82] who tested floating *Lemna minor* (L.) (Araceae) (Common duckweed), submerged *Potamogeton epihydrus* (Raf.) (Potamogetonaceae) (Ribbon-leaf pondweed), *Nuphar variegeta* (Durand.) (Nymphaeaceae) (Yellow pond-lily), and emerged *Typha latifolia* (L.) (Typhaceae) (Common cattail) in the removal of heavy metals in a mesocosm study. The study revealed that, amongst all macrophytes, floating macrophytes were more effective in assimilating heavy metals, followed by submerged, and lastly emergent macrophytes, which was similar to the present study.

Although there was promising heavy metal assimilation, the Swartkops River did not show overall water and habitat quality improvement downriver. This indicates that heavy metal reductions (>45%) in concentration between native and non-native macrophyte stands did not improve the water and sediment quality contamination; however, this was not the same for some important sediments and macrophyte pollution indices, which were variable across sites.

This could be due to constant influxes from multiple non-point and point sources (i.e., sewage treatment works, industries, and other anthropogenic activities) along the river system, meaning that the constant inputs have a significant effect on the system deterioration. Distance between sampled sites could have also influenced our findings, as some sites were located about one kilometre away from the non-native macrophyte stands, thus, allowing pollution inputs between sites, further suppressing the assimilation as seen in this study.

In addition, field experiments are considered dynamic and difficult to work with because they are complex and are affected by multiple extraneous variables that are not easy to control and can affect the outcome of results. Since this study was the first of its kind in the highly impacted Swartkops River system, we show that the phytoremediation technique can be effective; however, the state and land-use pressure play a crucial role, and we recommend more field-based studies with limited alterations.

## 7. Conclusions

The study showed the promising phytoremediation potential of native and non-native macrophytes to mitigate heavy metal contaminants from anthropogenic activities along the Swartkops River system. Water and sediment pollution indices were variable across sites showing no consistent trend in the reduction of water and sediment quality, and this was in contrast with our hypothesis. The lack of water and sediment quality improvement down river could have been due to constant pollution effluents from multiple non-point sources along the river system.

It is also possible that the river system could have been severely polluted to the extent that ecosystem services provided by both native and non-native macrophytes (although evident) were supressed. This study showed that native and non-native macrophytes can be used to assimilate pollutants; however, this can be better achieved in more control settings, i.e., laboratory and mesocosm settings, compared to complex and dynamic field conditions.

The screening of sediments and macrophytes (both native and non-native) provided an overview state of the Swartkops River system, and this may serve as an early warning or indication of changes in the system. Various authors [14,16,23,30,31,40] have demonstrated phytoremediation success in the reduction of water and sediments heavy metal concentrations; however, very few studies have tested if the improvement of water and sediment quality assists the recovery of biological diversity particularly through biological indicators, i.e., aquatic macroinvertebrates.

Thus, we propose that adjunctive studies should be conducted to assess phytoremediation using biological variables (periphyton, aquatic macroinvertebrate, etc.) to quantify phytoremediation success. The current study further emphasizes that physicochemical variables are not sensitive but variable and can only provide a snap-shot of habitat degradation. The sediment and macrophyte indices were reliable indicators of heavy metal contamination and macrophyte bio-accumulation potential; however, excessive anthropogenic input in the Swartkops River suppressed macrophyte ecosystem services. We therefore recommend more field studies to test various green technologies to mitigate the deterioration water and habitat quality using relevant biological indicators.

## Figures and Tables

**Table 1:** The geo-accumulation index (Igeo), enrichment factor (EF), and pollution load index (PLI) indices mean and ± standard deviation for measured heavy metals at 10 sites along the Swartkops River system over a period of six months (April 2018–September 2018). Bolded *H*-values indicate significant differences (Kruskal–Wallis ANOVA, *p <* 0.05).

Sediment Indices	Heavy Metals	Sites										*H*-Value
		1	2	3	4	5	6	7	8	9	10	
IGEO	As	2.94 ± 1.70	0.79 ± 0.89	−0.64 ± 1.71	1.15 ± 1.45	1.81 ± 0.81	1.53 ± 1.45	0.34 ± 0.57	0.81 ± 2.58	0.03 ± 1.22	0.26 ± 0.63	**17.16**
	Cd	−2.32 ± 2.15	−3.07 ± 3.03	−4.44 ± 2.87	−5.00 ± 3.62	−3.09 ± 2.88	−1.10 ± 2.46	−2.56 ± 3.15	−1.19 ± 2.67	−2.06 ± 2.86	−2.12 ± 2.94	8.26
	Cr	9.38 ± 0.44	9.76 ± 0.85	9.91 ± 0.85	9.69 ± 0.89	11.06 ± 0.86	9.77 ± 0.79	9.07 ± 0.55	9.29 ± 0.85	8.90 ± 0.91	8.85 ± 0.57	**19.08**
	Cu	5.60 ± 0.65	7.14 ± 0.23	6.41 ± 0.58	5.48 ± 1.37	7.02 ± 1.66	6.45 ± 1.26	4.41 ± 0.50	6.20 ± 0.57	5.38 ± 0.58	4.92 ± 0.21	**26.47**
	Fe	15.11 ± 0.86	14.67 ± 0.81	14.79 ± 0.45	13.80 ± 0.84	12.50 ± 0.96	13.97 ± 0.64	12.70 ± 1.11	14.33 ± 0.96	13.86 ± 0.61	13.91 ± 1.10	**24.32**
	Hg	−6.64 ± 3.63	−6.20 ± 2.52	−6.29 ± 2.03	−7.02 ± 2.09	−6.07 ± 2.09	−7.62 ± 3.05	−6.97 ± 1.65	−6.36 ± 1.71	−7.09 ± 2.52	−6.67 ± 1.78	5.05
	Pb	7.44 ± 0.62	7.61 ± 0.81	8.04 ± 0.60	6.75 ± 1.19	7.95 ± 0.58	5.33 ± 3.02	5.04 ± 3.15	6.29 ± 1.48	3.81 ± 3.56	3.66 ± 3.43	**26.19**
	Zn	8.77 ± 0.66	11.83 ± 0.64	11.37 ± 0.16	10.46 ± 1.12	12.16 ± 1.17	10.06 ± 0.93	9.06 ± 0.84	10.22 ± 0.93	9.44 ± 0.29	8.79 ± 0.36	**21.40**
EF	As	0.02 ± 0.01	0	0	0	0	0	0	0	0	0	**17.08**
	Cd	0.01 ± 0.01	0	0	0.01 ± 0.02	0	0	0	0	0	0	8.26
	Cr	0.01 ± 0.01	0	0	0	0	0	0	0	0	0	**20.39**
	Cu	0.01 ± 0.01	0	0	0	0	0	0	0	0	0	**26.47**
	Hg	1.09 ± 1.70	0.93 ± 1.31	0.66 ± 0.87	0.38 ± 0.46	0.71 ± 0.80	0.46 ± 0.76	0.33 ± 0.42	0.51 ± 0.62	0.52 ± 0.74	0.44 ± 0.58	1.76
	Pb	0.01 ± 0	0.01 ± 0	0.02 ± 0	0	0.02 ± 0	0.01 ± 0	0	0	0	0	**26.19**
	Zn	0	0.01 ± 0	0 ± 0	0	0.02 ± 0	0	0	0	0	0	**35.80**
PLI	April	0.86	0.63	0.43	0.25	1.10	0.00	0.00	0.00	0.00	0.00	9
	May	0.00	0.00	0.63	0.43	0.00	0.00	0.00	0.00	0.00	0.00	9
	June	0.19	0.38	0.69	0.00	0.56	0.64	0.00	0.40	0.46	0.00	9
	July	0.00	0.00	0.00	0.00	0.00	0.00	0.00	0.00	0.00	0.00	9
	Aug	0.52	0.00	0.90	0.98	0.85	0.00	0.00	0.00	0.00	0.00	9

**Table 2:** The bio-concentration factor (BCF) mean values and ± standard deviation for measured heavy metals from native and non-native macrophytes along 10 study sites in Swartkops River from April–September 2018. Bolded *H*-values indicate significant differences (Kruskal–Wallis ANOVA, *p* < 0.05). NB, *Pontederia crassipes* and *Stuckenia pectinata* were not present at site 1 throughout the study, hence, BCF values = 0 for all heavy metals.

Plant Species	Heavy Metals	Sites										*H*-Value
		1	2	3	4	5	6	7	8	9	10	
*T. capensis*	As	0.05 ± 0.07	0.25 ± 0.24	2.18 ± 2.89	0.39 ± 0.48	0.21 ± 0.12	0.37 ± 0.43	0.11 ± 0.24	1.55 ± 2.83	0.66 ± 1.25	0.08 ± 0.17	13.05
	Cd	0	0.07 ± 0.11	0.57 ± 0.611	1.10 ± 1.17	0.08 ± 0.09	1.75 ± 3.90	0.06 ± 0.13	0.03 ± 0.06	0.04 ± 0.09	0.05 ± 0.10	13.22
	Cr	0.65 ± 0.22	0.66 ± 0.58	0.50 ± 0.36	0.64 ± 2.91	0.23 ± 0.14	0.44 ± 0.22	0.44 ± 0.49	0.45 ± 0.32	0.57 ± 0.42	0.76 ± 0.63	8.88
	Cu	3.33 ± 1.77	1.32 ± 0.30	1.91 ± 0.70	3.86 ± 2.91	1.97 ± 2.97	1.93 ± 1.04	4.35 ± 1.74	1.99 ± 1.16	2.86 ± 0.90	3.87 ± 1.06	**21.11**
	Fe	0.34 ± 0.22	0.46 ± 0.28	0.39 ± 0.24	0.77 ± 0.65	2.10 ± 1.68	0.25 ± 0.08	0.76 ± 0.43	0.23 ± 0.16	0.41 ± 0.23	0.51 ± 0.38	16.06
	Hg	4.19 ± 6.65	0.76 ± 0.60	0.93 ± 0.98	1.16 ± 1.15	0.57 ± 0.31	3.82 ± 4.57	0.83 ± 0.78	1.12 ± 1.45	1.42 ± 2.23	0.72 ± 0.54	1.83
	Pb	0.08 ± 0.10	0.05 ± 0.05	0.04 ± 0.05	0.13 ± 0.12	0 ± 0.01	0.12 ± 0.17	0.19 ± 0.23	0.22 ± 0.30	0.07 ± 0.10	0.09 ± 0.13	2.76
	Zn	2.21 ± 1.01	0.24 ± 0.10	0.33 ± 0.07	0.76 ± 0.84	0.24 ± 0.25	1.81 ± 1.22	5.47 ± 2.79	2.34 ± 3.05	2.50 ± 0.89	4.18 ± 1.86	**37.34**
*C. sexangularis*	As	0.05 ± 0.06	0.25 ± 0.24	2.35 ± 2.95	0.39 ± 0.48	0.23 ± 0.19	0.48 ± 0.63	0.11 ± 0.24	1 ± 1.75	0.48 ± 0.52	0.08 ± 0.18	11.20
	Cd	0.21 ± 0.35	0.12 ± 0.22	0.37 ± 0.60	1.10 ± 1.17	0.22 ± 0.34	0.14 ± 0.32	0.06 ± 0.13	0.04 ± 0.08	0.03 ± 0.07	0.05 ± 0.10	11.02
	Cr	0.57 ± 0.16	0.55 ± 0.38	0.58 ± 0.50	0.64 ± 0.35	0.21 ± 0.12	0.52 ± 0.32	0.68 ± 0.52	0.60 ± 0.55	0.65 ± 0.47	0.98 ± 0.47	11.49
	Cu	2.34 ± 1.50	1.73 ± 0.21	2.21 ± 0.59	3.86 ± 2.91	2.13 ± 2.90	2.30 ± 2.11	4.35 ± 1.74	1.24 ± 0.41	2.59 ± 2.29	3.87 ± 1.06	**25.39**
	Fe	0.38 ± 0.20	0.28 ± 0.14	0.39 ± 0.23	0.77 ± 0.65	2.37 ± 2.17	0.67 ± 0.44	0.76 ± 0.43	0.24 ± 0.13	0.31 ± 0.09	0.51 ± 0.38	15.87
	Hg	0.30 ± 0.13	0.471 ± 0.55	0.88 ± 0.85	1.16 ± 1.15	0.66 ± 0.41	2.64 ± 2.68	0.83 ± 0.78	0.48 ± 0.39	1.04 ± 1.57	0.72 ± 0.54	10.10
	Pb	0.09 ± 0.09	0.05 ± 0.05	0.05 ± 0.05	0.13 ± 0.12	0.06 ± 0.08	0.08 ± 0.10	0.19 ± 0.23	0.16 ± 0.14	0.09 ± 0.10	0.09 ± 0.13	3.77
	Zn	4.74 ± 2.59	0.97 ± 0.56	0.29 ± 0.06	0.76 ± 0.84	0.28 ± 0.32	0.86 ± 0.40	5.47 ± 2.79	2.45 ± 1.28	3.75 ± 0.91	4.18 ± 1.86	**38.49**
*P. australis*	As	0.10 ± 0.08	0.11 ± 0.16	1.75 ± 2.44	0.14 ± 0.31	0.09 ± 0.20	0.18 ± 0.30	0.01 ± 0.03	0.08 ± 0.11	0.15 ± 0.20	0.09 ± 0.20	4.25
	Cd	0.19 ± 0.41	0.072 ± 0.11	0.98 ± 1.72	2.67 ± 5.29	0.02 ± 0.04	0	0	0	0	0.07 ± 0.15	15.76
	Cr	0.59 ± 0.41	0.29 ± 0.24	0.25 ± 0.17	0.25 ± 0.19	0.10 ± 0.18	0.53 ± 0.40	0.63 ± 0.53	0.64 ± 0.52	0.86 ± 0.63	0.70 ± 0.79	12.02
	Cu	1.38 ± 0.73	0.58 ± 0.34	0.89 ± 0.20	1.41 ± 0.97	0.47 ± 0.42	1.85 ± 1.94	2.06 ± 1.65	0.86 ± 0.64	1.82 ± 1.52	1.97 ± 1.34	11.73
	Fe	0.38 ± 0.28	0.36 ± 0.34	0.46 ± 0.36	1.30 ± 1.74	0.88 ± 1.17	0.33 ± 0.31	0.54 ± 0.48	0.14 ± 0.13	0.47 ± 0.66	0.38 ± 0.16	9.58
	Hg	0.31 ± 0.18	0.53 ± 0.58	0.64 ± 0.81	0.60 ± 0.54	0.28 ± 0.47	1.46 ± 1.37	0.91 ± 1.28	0.51 ± 0.61	1.47 ± 2.49	0.78 ± 0.67	4.12
	Pb	0.07 ± 0.07	0.04 ± 0.05	0.04 ± 0.06	0.07 ± 0.10	0	0.04 ± 0.09	0.09 ± 0.13	0.07 ± 0.10	0.03 ± 0.08	0.09 ± 0.13	2.30
	Zn	3.70 ± 1.72	1.40 ± 1.00	1.67 ± 1.13	2.36 ± 2.72	0.63 ± 0.57	3.27 ± 2.55	2.56 ± 1.84	1.56 ± 1.13	6.53 ± 9.05	6.18 ± 4.23	**16.43**
*P. crassipes*	As	0	0.15 ± 0.16	0	0	0.13 ± 0.12	0.23 ± 0.51	0	1.54 ± 2.64	1.08 ± 1.34	0.10 ± 0.17	**23.15**
	Cd	0	1.18 ± 2.61	0.09 ± 0.15	0.25 ± 0.43	0.41 ± 0.86	0.03 ± 0.07	0.07 ± 0.15	0.04 ± 0.08	0.03 ± 0.07	0.06 ± 0.13	4.28
	Cr	0	0.45 ± 0.21	0.35 ± 0.22	0.40 ± 0.20	0.13 ± 0.07	0.29 ± 0.14	0.37 ± 0.28	0.51 ± 0.28	0.82 ± 0.42	0.80 ± 0.62	**28.32**
	Cu	0	1.70 ± 0.66	2.79 ± 0.99	7.10 ± 7.08	3.43 ± 4.21	1.99 ± 1.38	8.01 ± 4.07	2.23 ± 0.68	3.93 ± 2.60	4.86 ± 3.09	**24.40**
	Fe	0	0.52 ± 0.29	0.41 ± 0.24	0.86 ± 0.63	1.88 ± 0.99	0.27 ± 0.15	0.77 ± 0.54	0.81 ± 0.73	0.98 ± 0.64	1.15 ± 0.95	**29.94**
	Hg	0	0.41 ± 0.36	0.44 ± 0.85	0.55 ± 1.08	0.33 ± 0.24	1.25 ± 1.21	1.29 ± 1.48	0.59 ± 0.52	1.35 ± 2.19	1.10 ± 0.78	**20.76**
	Pb	0	0.11 ± 0.12	0.01 ± 0.01	0.04 ± 0.07	0.08 ± 0.08	0.09 ± 0.12	0.21 ± 0.25	0.24 ± 0.33	0.09 ± 0.11	0.21 ± 0.24	12.82
	Zn	0	2.46 ± 2.08	1.35 ± 0.51	2.95 ± 2.44	1.65 ± 1.68	3.36 ± 1.25	8.34 ± 5.74	2.32 ± 1.18	4.47 ± 1.93	5.94 ± 3.38	**27.70**
*S. pectinata*	As	0	0.23 ± 0.28	2.69 ± 4.51	0.29 ± 0.29	0.18 ± 0.18	0.38 ± 0.57	0.22 ± 0.34	2.88 ± 6.40	0.44 ± 0.70	0.20 ± 0.33	11.42
	Cd	0	0.14 ± 0.22	0.18 ± 0.28	0.49 ± 0.85	0.13 ± 0.17	0	0.15 ± 0.33	0.07 ± 0.16	0.06 ± 0.13	0.05 ± 0.12	13.37
	Cr	0	0.71 ± 0.54	0.64 ± 0.47	0.74 ± 0.44	0.65 ± 0.33	1.58 ± 0.88	1.60 ± 0.64	0.99 ± 0.77	1.60 ± 1.44	0.97 ± 0.37	**27.33**
	Cu	0	112.32 ± 141.42	83.46 ± 76.84	178.19 ± 262.57	112.59 ± 117.41	809.31 ± 1561.55	52.59 ± 45.09	33.02 ± 27.44	54.84 ± 65.20	19.88 ± 22.27	15.11
	Fe	0	2.01 ± 2.34	1.54 ± 1.68	3.79 ± 5.46	11.76 ± 8.44	3.26 ± 1.87	18.77 ± 17.96	2.70 ± 1.81	3.47 ± 2.15	3.96 ± 4.83	**23.64**
	Hg	0	13.17 ± 17.67	9.38 ± 10.54	19.85 ± 30.46	9.40 ± 10.92	71.04 ± 128.50	6.87 ± 9.13	1.13 ± 1.18	7.19 ± 8.81	2.09 ± 2.51	**16.77**
	Pb	0	0.31 ± 0.10	0.23 ± 0.09	0.61 ± 0.35	0.28 ± 0.10	0.52 ± 0.36	0.70 ± 1.04	0.49 ± 0.47	0.40 ± 0.45	0.48 ± 0.63	13.94
	Zn	0	5.79 ± 2.63	5.60 ± 1.80	10.99 ± 5.59	5.41 ± 6.69	18.95 ± 13.17	15.86 ± 16.06	4.42 ± 3.00	8.73 ± 1.35	11.22 ± 4.40	**22.08**

## Supplementary Materials

The following are available online at https://www.mdpi.com/article/10.3390/plants10122676/s1, Table S1: A summary of bio-physical characteristics of the ten sampling sites at the Swartkops River system, Eastern Cape, South Africa. Table S2: Water chemistry mean values and ±standard deviation recorded from 10 sites, including non-native macrophytes stands along the Swartkops River system South Africa from April–September 2018. Bolded *H*-values indicate significant differences (Kruskal-Wallis ANOVA, *p* < 0.05); NS = not significant, *p* > 0.05. Table S3: Sediment chemistry mean and (±standard deviation) recorded from 10 sites, including native macrophytes stands along the Swartkops River system South Africa (April 2018–September 2018). Bolded *H*-values indicate significant differences (Kruskal-Wallis ANOVA, *p* < 0.05). Table S4: Percentage reduction of heavy metals concentration in sediments semi and permanent stands of *Pontederia crassipes* and *Salvinia molesta* along the Swartkops River system, Eastern Cape, South Africa.

## Novelty Statement

Considering the alarming effects of anthropogenic activities on water quality and biodiversity in freshwater systems, our study assessed the assimilation potential of native and non-native macrophytes in a potentially toxic urban freshwater system through phytoremediation. This study is amongst the few studies that have tested phytoremediation in a field setting (in-situ), providing crucial information associated with quantifying phytoremediation in field settings. The findings from our study clearly showed the promising assimilation of both native and non-native macrophytes, and we strongly believe that our insights from this study will promote the use of macrophytes to mitigate pollution and restore riverine systems.
