# Supplementary material for: Heavy Metals Assimilation by Native and Non-Native Aquatic Macrophyte Species: A Case Study of a River in the Eastern Cape Province of South Africa
Source: Plants (Basel). 2021 Dec 6;10(12):2676. doi: 10.3390/plants10122676 (PMC8703371; doi:10.3390/plants10122676)
Supplement: Supplementary file 1 [file plants-10-02676-s001.zip › plants-1448168-Supplementary.pdf]

Supplementary Materials

Table S1. A summary of bio-physical characteristics of the ten sampling sites at the Swartkops River system, Eastern Cape, South Africa.

Study sites	Riparian vegetation	Stream vegetation	Site structure and characteristics
1	Grasses and a few shrubs	*Cyperus* spp., *Phragmites* spp., and *Typha* spp.	Open canopy, shallow, narrow channel composed of cobbles and a few pebble stones, with a medium flow and high water clarity
2	*Typha* spp., and dense *Phragmites* spp.	*Nasturtium officinale,* and *Stuckenia pectinata*	Open canopy, composed of pebbles and boulders with medium turbidity.
3	*Typha* spp., and dense *Phragmites* spp.	Permanent *P. crassipess* dense mat and *S. pectinata*	Open canopy, composed of pebbles and boulders with medium turbidity
4	*Typha* spp., and dense *Phragmites* spp.,	*N. officinale* and *S. pectinata*	Open canopy, composed of pebbles, boulders and deep waters
5	Dense *Phragmites* spp., *Cyperus* spp., *Typha* spp., and *Eucalyptus* spp.	*N. officinale and S. pectinata*	Wider river channel, shallow water depth, open canopy. Composed of cobbles and boulders. Turbid and occasionally higher water velocity caused by sewage effluent
6	*Phragmites* spp., *Cyperus* spp., *Typha* spp., and *Eucalyptus* spp.	Permanent *P. crassipess* dense mat	Open canopy, fully dense *P. crassipe*s cover on the water column
7	*Phragmites* spp., *Cyperus* spp., *Typha* spp., and *Eucalyptus* spp.	*N. officinale* and *S. pectinata*	Wider river channel, shallow water depth, open canopy. Composed of cobbles and boulders
8	*Eucalyptus* spp., and *Typha* spp.	*N. officinale* and *S. pectinata*	Open canopy, composed of cobbles and pebbles, shallow to deep waters

9	*Eucalyptus* spp., and *Typha* spp.	*S.molesta* dense mat and *S. pectinata*	Open canopy, deep waters, and few cobbles
10	*Eucalyptus* spp., and *Typha* spp.	*N. officinale* and *S. pectinata*	Open canopy, shallow waters, cobbles and pebbles

Table S2. Water chemistry mean values and ± standard deviation recorded from 10 sites, including non-native macrophytes stands along the Swartkops River system South Africa from April – September 2018. Bolded *H*-values indicate significant differences (Kruskal-Wallis ANOVA, $p< 0.05$); NS = not significant, $p > 0.05$.

	**Sites**										
**Heavy metals**	**1**	**2**	**3**	**4**	**5**	**6**	**7**	**8**	**9**	**10**	***H*-Value**
Fe (mg/l)	1.1±0.45	0.19±0.07	0.2±0.05	0.18±0.05	0.24±0.14	0.13±0.10	0.14±0.06	0.10±0.03	0.13±0.10	0.09±0.02	**28.138**
Cu (mg/l)	0.02±0.02	0.02±-0.01	0.02±0.01	0.04±0.04	0.4±0.04	0.02±0.02	0.02±0.01	0.02±0.01	0.02±0.02	0.02±0.01	1.965
Zn (mg/l)	0.02±0	0.02±0	0.02±0	0.02±0	0.04±0.02	0.02±0	0.03±0	0.02±0	0.02±0	0.12±0.23	**18.083**
COD (mg/l)	14.64±13.39	28.6±10.92	37.2±7.5	28±12.67	57.4±22.51	55.8±18.51	46±25.09	32±10	36±17.74	30.2±10.99	**21.892**
As (µg/L)	4±0	5.72±2.44	49±0	5±2.24	9±9.40	4±0	4±0	4±0	6.49±2.73	4±0	16.532
Cd (µg/L)	2.1±0	2.1±0	2.1±0	2.1±0	2.1±0	2.1±0	2.1±0	2.1±0	2.1±0	2.1±0	NS
Cr (µg/L)	26±0	26±0	26±0	26±0	26±0	26±0	26±0	26±0	26±0	26±0	NS
Hg (µg/L)	2.1±0	2.1±0	2.1±0	2.1±0	2.1±0	2.1±0	2.1±0	2.1±0	2.1±0	2.1±0	NS
Pb (µg/L)	6±0	6±0	6±0	6±0	6±0	6±0	6±0	6±0	6±0	6±0	NS

Table S3. Sediment chemistry mean and (± standard deviation) recorded from 10 sites, including native macrophytes stands along the Swartkops River system South Africa (April 2018–September 2018). Bolded *H*-values indicate significant differences (Kruskal–Wallis ANOVA, $p < 0.05$).

	Sites										
**Heavy metals (mg/kg)**	**1**	**2**	**3**	**4**	**5**	**6**	**7**	**8**	**9**	**10**	***H*-Value**
Fe	1321.25±838.89	967.37±641.5	950.78±283.05	520.02±261.10	220.43±136.60	554.19±230.32	268.95±206.10	769.73±421.7	511.56±210.11	628.36±514.10	**24.32**
Zn	7.59±4.27	62.55±31.04	41.92±4.05	26.92±15.30	87.16±45.38	19.89±12.44	9.65±5.63	22.4±15.97	11.17±2.24	7.19±1.92	**35.79**
Cd	0.21±0.21	0.12±0.15	0.14±0.16	0.17±0.32	0.10±0.11	0.02±0.05	0.02±0.03	0.02±0.04	0.06±0.10	0.06±0.09	8.260
As	4±2.67	0.61±0.68	0.32±0.42	1.20±1.44	1.49±0.83	1.46±0.10	0.28±0.42	1.55±1.90	0.42±0.42	0.27±0.43	**17.08**
Cr	11.54±3.37	16.23±6.98	18.03±7.53	16.02±10.28	41.12±25.25	16.14±7.34	9.50±3.59	11.73±5.21	9.08±4.32	8.15±2.86	**20.39**
Pb	14.07±6.73	16.36±7.50	21.10±7.39	10.23±7.24	19.90±8.54	6.43±4.51	6.45±5.57	8.42±6.79	4.32±5	3.67±4.41	**26.19**
Hg	2.07±3.25	1.77±2.50	1.26±1.48	0.72±0.88	1.36±1.52	0.87±1.45	0.64±0.60	0.98±1.19	0.99±1.41	0.83±1.11	1.756
Cu	1.58±0.75	4.29±0.67	2.73±1.01	1.83±1.49	5.56±3.96	3.43±2.47	0.67±0.24	2.34±0.86	1.32±0.45	0.92±0.14	**26.46**
P	296.24±128.88	978.45±410.0	489.94±275.31	391.60±543.64	2240.83±1899.70	454.77±333.68	281.07±215.38	1077.85±274.5	863.70±542.90	468.8±344.05	**21.63**

Table S4. Percentage reduction of heavy metals concentration in sediments semi and permanent stands of *Pontederia crassipes* and *Salvinia molesta* along the Swartkops River system, Eastern Cape, South Africa.

	**Percentage reduction between immediate upstream and downstream sites per non-native macrophyte patch**		
**Heavy metals in sediments**	**Non-native macrophyte 1 (*P. crassipes*)**	**Non-native macrophyte 2 (*P. crassipes*)**	**Non-native macrophyte 3 (*S. molesta*)**
Fe	46	No reduction	18
Zn	57	89	65
As	No reduction	81	83
Cr	1	77	31
Pb	37	68	56
Hg	59	53	15
Cu	57	88	60
P	60	87	57
